# Functionalization of Alpha-Lactalbumin by Zinc Ions

**DOI:** 10.1021/acsomega.2c03674

**Published:** 2022-10-21

**Authors:** Adrian Gołębiowski, Paweł Pomastowski, Katarzyna Rafińska, Petar Zuvela, Ming Wah Wong, Oleksandra Pryshchepa, Piotr Madajski, Bogusław Buszewski

**Affiliations:** † Centre for Modern Interdisciplinary Technologies, Nicolaus Copernicus University in Torun, 4 Wileńska Street, 87-100 Torun, Poland; ‡ Department of Environmental Chemistry and Bioanalytics, Faculty of Chemistry, Nicolaus Copernicus University in Torun, 7 Gagarina Street, 87-100 Torun, Poland; § Department of Chemistry, National University of Singapore, 3 Science Drive 3, 117543 Singapore, Singapore; ∥ Department of Chemistry of Materials Adsorption and Catalysis, Faculty of Chemistry, Nicolaus Copernicus University in Torun, Gagarina 7, 87-100 Torun, Poland

## Abstract

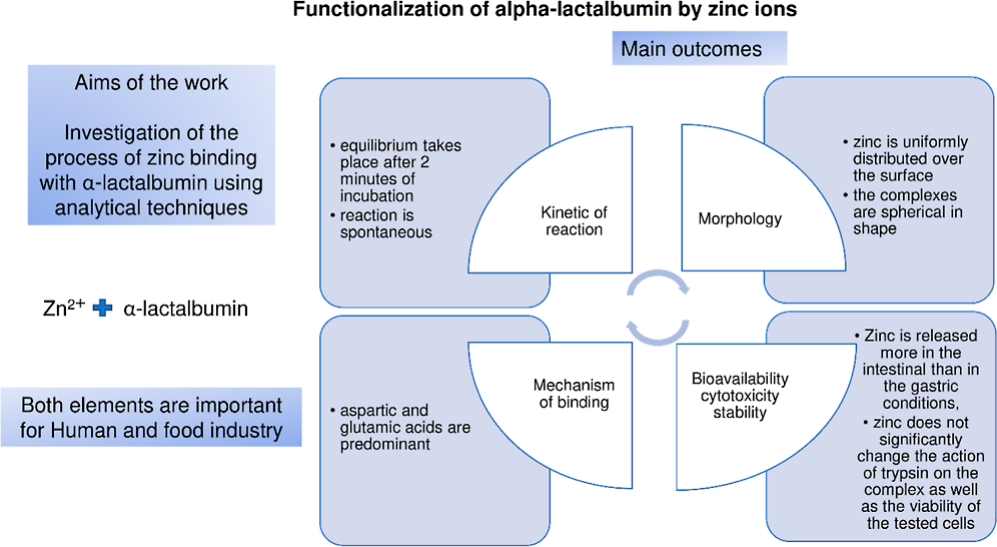

Alpha-lactalbumin (α-LA) and binding of zinc cations to protein were studied. Molecular characteristics of protein was determined by MALDI-TOF/MS and electrophoresis SDS-PAGE, and also, for complexes, it was determined by spectroscopic techniques (ATR-FT-IR and Raman) and microscopic techniques (SEM along with an EDX detector and also TEM). The pH dependence of zeta potential of α-LA was determined in saline solution. The zinc binding to the protein mechanism was investigated; zinc binding to protein kinetics, the molecular modeling by the DFT method, and electron microscopy (SEM and TEM) for microstructure observation were performed. The experiments performed indicate a quick binding process (equilibrium takes place after 2 min of incubation) which occurs onto the surface of α-LA. Zinc cations change the conformation of the protein and create spherical particles from the morphological point of view. DFT studies indicate the participation of acidic functional groups of the protein (aspartic acid and glutamic acid residues), and these have a decisive influence on the interaction with zinc cations. Application studies of general toxicity and cytotoxicity and bioavailability were conducted.

## 1 Introduction

Alpha-lactalbumin (α-LA) is a protein, which constitutes 123 amino acids and has a molar mass of the monomer of about 14 kDa.^1^ It is the most relevant milk protein in humans (about one-quarter of mass). It is present in all mammalian species, but the content differs in individual organisms.^2,3^ Protein is a part of lactase synthetase, and its source of relevant amino acids, consequently, plays a key role in nutrition of organisms, especially infants.^4,5^ The structural similarity to lysosomes is noted, but proteins have different properties and play different roles in living organisms.^6^ The α-helix has the highest content in the overall conformation of a protein in its native state and undergoes a self-cross-linking reaction.^7^ α-LA occurs as holo and apo forms, and calcium ions are tightly bound (which appear as the holo form) to the calcium binding site.^8^ Protein has also secondary binding sites.^9^ The properties of protein are strictly depending on the protein form. The holo form of α-LA (calcium-bound state) increases the stability of protein,^6,10,11^ although the apo form of protein gives higher opportunity to bind other metal ions.^12,13^ However, other ions such as sodium, divalent, and trivalent ions compete with each other to binding sites of α-LA.^14,15^ This protein is one of the most acidic proteins (the isoelectric point is from 4.2 to 4.6).^16^ In differentiation to beta-lactoglobulin, the main protein in bovine milk, α-LA, does not have free thiol residues, and consequently, at elevated temperature and under denaturated conditions, it does not create a gel structure.^17^ The characteristic property of α-LA is creating the molten globule state of protein under acidic conditions or using specific denaturated conditions.^18^ Protein in this state is known to be a specific transport agent and can possess lethal activity to tumor cells. The HAMLET (human α-LA makes lethal to tumor cells) and BAMLET (bovine α-LA makes lethal to tumor cells) approaches are extensively studied as anti-tumor agents.^19,20^ α-LA protein has the ability to bind trace amounts of metals (Zn, Fe, Mn, *etc.*) and release them during digestion in the gastrointestinal tract. This is especially important for the supply of micronutrients during the nutrition of infants, in particular.^21^ Therefore, research on the synthesis and characterization of this type of connection, such as protein micronutrients (zinc), is important from a scientific and industrial point of view. In addition, the synthesis mechanism (especially the participation of individual amino acids) and the action and factors affecting the characteristics of this type of complexes have not yet been fully understood.

Zinc is noted as a *trans*-metal element. Zinc has five isotopes (64, 66, 67, 68, and 70 amu) which make up an average molecular weight of 65.38 units. In consideration of the chemical activity, it is a reducing agent, and in biological fluids, it exhibits a double valence state. In solution, the zinc ion is hydrated, and the coordination compounds are created. Zinc ions are coordinated commonly by six water molecules.^22^ Zinc ions are one of the necessary micronutrients. It is found in all plants and animals, which may prove its popularity. It is essential in maintaining homeostasis and the proper functioning of the immune system and growth; however, excessive supplementation has negative effects similar to the deficiency of an element in the body;^23^ however, zinc in the body is participating in signal transduction as a second messenger.^24^ It is a cofactor of many enzymes, and it constitutes catalytic activity in many biological reactions.^25^ Thus, taking protein–zinc complexes can promote healthy growth of all humans.^26^ A zinc ion possesses affinity to oxygen and sulfur atoms especially. Thus, zinc creates complexes with α-LA due to interaction with amino acid moieties: glutamic and aspartic acids and also histidine.^6^ There are two sets of binding places on the surface of the protein. The first was a bond of the order 10^4^ to 10^5^, and the second was a bond of approximately 10^3^.^27,28^ Binding of metal to protein occurs gradually in relation to the molar ratio of protein to zinc. The zinc–protein complex shows lower thermal stability than the holo protein form, leading, in consequence, to aggregation and higher susceptibility to digestion with proteolytic enzymes.^27^ The bovine α-LA structure and its complexes with zinc were studied using the techniques of circular dichroism and nuclear magnetic resonance. The binding of zinc to the holo form did not lead to large structural changes but to small local changes only.^11^ Single and slight structural changes of the protein in the form of apo and holo after binding with zinc were noticed using the Fourier transform infrared spectroscopy (FT-IR) technique.^10^

The zinc complexes of α-LA are not yet fully understood. There is a lack of extensive research into the in-depth characterization of both the protein and the zinc complex. Therefore, the aim of this work was to investigate α-LA and protein–zinc complexes. Multidisciplinary research was carried out using advanced instrumental and computational simulations techniques (MALDI-TOF-MS/MS, PAGE, ATR-FT-IR and Raman spectroscopy, physicochemical stability in solution by zeta potential determination, kinetic study of zinc binding using ICP–MS to trace zinc concentration determination, complex stability in synthetic physiological fluids, morphology studies by SEM, TEM, and molecular dynamics simulation by DFT calculations). Extended knowledge of this type of connection can be valuable in understanding and describing the mechanism of the formation of the α-LA–Zn complexes and using this knowledge for supplementation both as a source of endogenic protein and using it as a carrier of the necessary minerals.

## 2 Materials and Methods

The commercial standard of α-LA was used in all experiments. The protein was bought from Sigma-Aldrich (Sigma-Aldrich, Steinheim, Germany). The supplier states that the purity of the material is higher than 85% (SDS-PAGE method).

### 2.1 Characterization of α-LA by SDS-PAGE

The purity of the product and molar mass of protein were assessed by applying the SDS-PAGE technique using a method adopted from ref (29) with modifications. Electrophoresis was performed using the Thermo Scientific apparatus (Thermo Scientific, Waltham, MA, USA). The used gel was Invitrogen Bolt 4–12% Bis-Tris Plus (Thermo Scientific, Waltham, MA, USA). The markers of protein mass were SeeBlue Plus2 Pre-Stained Standard (Thermo Scientific, Waltham, MA, USA). The gel was stained using the Coomassie Blue method. The protein solution of about 2 mg/mL was prepared in double-deionized water. Two additional 10-fold serial dilutions of stock samples were prepared. The reduced and nonreduced modes were utilized. Samples were prepared according to a manufacturer (Invitrogen) procedure. Briefly, protein solution was dispersed in a 2.5 μL load sample buffer (LDS). Reduction and alkylation were prepared using the sample reducing agent (10×)—dithiothreitol (DTT) and iodoacetamide (IAA), respectively. The samples were then heated for 10 min at 70 °C and introduced to the gel. Nonreduced samples were prepared without the last step (reduction and alkylation). Running buffer was MES. The electrophoresis process was executed at a voltage of 200 V. After the separation process, the gel was stained for 20 min. The discoloration was carried out at least for 24 h in double-deionized water at room temperature.

### 2.2 Characterization of α-LA by the MALDI-TOF/TOF MS Technique

The MALDI-TOF/TOF MS technique in the linear positive mode (*intact*) for molar mass determination and in the reflectron positive mode for investigation of peptide fingerprint mass spectra (PMF) after the protein digestion procedure with trypsin were used. The method^29,30^ was adopted with modifications. A MALDI-TOF/TOF mass spectrometer (Bruker Daltonics, Bremen, Germany) equipped with a modified Nd:YAG laser operating at a wavelength of 355 nm and frequency of 2 kHz was used. The reagents were purchased from Sigma-Aldrich (Steinheim, Germany) with the highest commercially available degree of purity. The α-cyano-4-hydroxycinnamic acid (HCCA) was used as a matrix in the reflectron mode, while 3,5-dimethoxy-4-hydroxycinnamic acid (sinapic acid—SA) was used in the linear mode (all from Bruker Daltonics, Bremen, Germany). The calibration was carried out applying Peptide Calibration Standard II and Protein Calibration Standard II all from Bruker Daltonics (Bremen, Germany) for PMF and intact analyses, respectively. The dried droplet method was applied in intact analysis, while for PMF analysis, the Bruker Proteomic protocols for mass spectrometry were applied.^31^ The MS spectra of α-LA intact were recorded in the range of *m*/*z* 5000–50,000, while the peptide fingerprint mass spectra (PMF) of protein digested with trypsin were recorded in the range of *m*/*z* 700–3500. In both cases, the measurements were carried out at an accelerating voltage of 25 kV. To determine the fragmentation spectra, the laser-induced fragmentation technique (LIFT) in the same *m*/*z* range was used. The peptides obtained after the tryptic digestion of α-LA were identified using BioTools software (Bruker Daltonics, Bremen, Germany). All data were collected manually, and the mass tolerance was set to 0.3 Da for the spectra and calibrated internally on immonium ions at a laser power of 60% and an attenuation of 27% for the MS/MS analysis.

### 2.3 Characterization of α-LA Saline Using Laser Doppler Velocimetry and Phase Analysis Light Scattering (PALS) (M3-PALS)

Zeta potential values were determined in the pH range from 2 to 8. α-LA solution (0.4 mg/mL) was prepared in 0.09% (w/v) NaCl. The method was adopted from refs (29) and (30) with modifications. After protein was dissolved, the pH was adjusted to a certain value against the pH meter (1 M HCl and 1 M NaOH solutions were used to correction) (first, solution at a pH of 2 was prepared). A portion of solution was loaded to the DTS 1070 cuvette (Malvern, Worcestershire, UK), and zeta potential was determined using the Malvern Zetasizer NanoZS apparatus (Malvern, Worcestershire, UK). After completion of measurement, the sample was turn back to stock protein solution. The solution was used again; the solution was mixed with the remaining solution, and another sample was prepared (pH correction and application to the cuvette). Protein solution was adjusted to a higher pH by dropwise addition of acid or alkali solution to reach the pH about 0.5 units higher than that of the previous sample. Smoluchowski’s approximation in Henry’s equation was used. Measurements were performed at room temperature, with automatic selection of voltages and the number of runs for software. For the result, three replications of run were averaged. To process data, the sigmoidal model was used.

### 2.4 Synthesis of α-LA Complexes with Zinc; Kinetic Study of Zinc Binding to Protein by the ICP–MS Technique

#### 2.4.1 General Synthesis Method

The sample preparation protocol was adopted from refs (30) and (32) modifications. The protein stock solution with a concentration of 5 mg/mL was prepared in pH 4.5 in 0.09% (w/V) NaCl solution. The pH was adjusted to pH of the protein isoelectric point (pH 4.5). Zinc solution with a concentration of 60 mg/L was prepared under the same conditions as a protein from the nitrate salt (Sigma-Aldrich, Steinheim, Germany). The solutions were mixed in the volume ratio 1:1.

#### 2.4.2 Kinetic Study of Zinc Binding

The samples were incubated at room temperature by constantly stirring at 900 rpm on a Thermomixer. Times of incubation were 2, 5, 10, 20, and 30 min and 1, 2, 4, and 6 h. After reaching incubation time, the unbound metal fraction solution was separated on Amicon Ultracell 3 kDa (Merck, Darmstadt, Germany). The solution was centrifuged for 15 min at 4 °C, 14,000 rpm. The filtrate solution was diluted (dilution factor 200) in 1% HNO_3_ solution (Suprapure grade) (Merck, Darmstadt, Germany). The initial zinc concentration was determined as well. The calibration curve method was used to obtain the concentration result in samples. Zinc standard solution (Sigma-Aldrich, Steinheim, Germany) was diluted to the appropriate region of concentrations. Scandium was used as an internal standard. In this research, Shimadzu ICPMS 2030 (Shimadzu, Kyoto, Japan) was used. The collision reaction cell with the helium mode was used. The signals at *m*/*z* of 66 and 67 were monitored. The binding kinetics was determined as the difference between the initial zinc concentration and not bound fraction of zinc by the protein. The experimental data were analyzed using zero-order, pseudo-zero kinetics, and Weber–Morris intraparticle models. The fitting was performed by the least-squares method. Also, the thermodynamic parameters such as the amount of zinc bound to protein *Q*_t_, distribution coefficient *K*_d_, and Gibbs free energy of adsorption were displayed.^32,33^

### 2.5 Preparation of Complexes for Further Studies

According to the kinetic study, the complex sample was incubated for about 10 min. The unbound metal fraction was separated as in 2.4. Section. The supernatant was excluded, the remaining solution was centrifuged (14,000 rpm, 20 °C) using the Amicon 3 kDa membrane (Merck, Darmstadt, Germany), and the pellet was washed twice with deionized water. The solution was recovered from the membrane. The complexes were lyophilized (FreeZone Labconco, Kansas City, US). Dried complexes were stored at −20 °C.

### 2.6 Characterization of α-LA Complexes with Zinc by Spectroscopic Techniques (ATR-FTIR and Raman)

The sample was probed to characterization by attenuated total reflection Fourier transform infrared spectroscopy (ATR-FTIR) using an Alpha FTIR spectrometer apparatus (Bruker, Billerica, Massachusetts, USA).^30,32^ Spectra were obtained in the range 400–4000 cm^–1^. The dried sample was attached to the measurement window.

Raman spectra were recorded using a Raman spectrometer (Senterra, Bruker Optik).^30,32^ The protein was dissolved in small volume of water. A tiny droplet of suspension was injected to glass. The spectra were recorded in the region 4000–400 cm^–1^ at the wavelength 532 nm as excitation light, with a power of approximately 20 mW, and the spectrum was counted two times at 30 s. The spectroscopic data were processed with OPUS software.

### 2.7 Characterization of α-LA Complexes with Zinc by Microscopic Techniques: SEM, SEM–EDX, and TEM

To obtain information about morphology, topography, and quantitative analysis of elements in protein and complexes, analysis using scanning electron microscopy (SEM) along with EDX was carried out.^30,32^ The apparatus used were as follows: SEM (Quanta 3D) and SEM–EDX instrument [1430 VP (LEO Electron Microscopy Ltd, UK)]. The dried powder was applied on the carbon tape.

TEM microscopy gives higher resolution and deeper view into the morphological structure. The dried material of complexes was dispersed in anhydrous ethanol and applied on a carbon lacey copper grid. Measurements were performed using the TEM apparatus (model G2 F20X-Twin 200 kV, FEI).

### 2.8 Characterization of α-LA Complexes with Zinc by Molecular Dynamics

Molecular dynamics (MD) study was performed according to the protocol of Pomastowski *et al.*(34) and Žuvela *et al.*(35) The α-LA was analyzed in the apo form, and their complexes with zinc were characterized. Solvation in a TIP3P water box with a variable side length, depending on the size of the system, was performed. Due to the limited volume of solvation boxes, the amounts (in moles) of α-LA–Zn solutions were downscaled with constant scaling factors (to fully preserve the concentration ratios for Zn−α-LA); from initial zinc concentration of 30 mg/L in mixture, the n(ions) per protein. molecule is 2.57 and scaled of 39.

Structures of the proteins used for the computational characterization are as in ref (36). α-LA was characterized with its holo form (Ca^2+^ cation bound within its structure). The native ions were preserved in all the APO structures and their complexes since they define the protein functions. To account for nonbonded interactions of Zn^2+^ with α-LA, parameters compatible with the TIP3P water model were obtained from Li *et al.*;^37,38^ briefly, *R*_min_/2 is 1.271, epsilon is 0.00330286, and sigma is 0.226466.

Electrostatic neutralization with Na^+^ or Cl^–^ ions, EM to remove bad contacts and structural clashes, heating to 298.15 K at a constant volume, and equilibration of density by subjecting systems to constant were carried out. A pressure of 1 bar and a temperature of 298.15 K—*NPT* ensemble—were used; production MD simulations in the *NVT* ensemble were used. MD simulations were carried out using GROMACS 5.1.2 software using the AMBER ff99-SB-ILDN force field.^39^ Visual Molecular Dynamics (VMD) 1.9.3 software and Python 3.9 were used for visualization and data analysis.

### 2.9 Binding Interaction of Zn^2+^ with Aspartate and Glutamate Residues by DFT Calculation

To shed light on the interaction of the zinc ion (Zn^2+^) and α-LA protein, density functional theory (DFT) calculations, using Gaussian 16 Programs,^40^ were carried out to examine various possible 1:1 Zn^2+^–Asp^–^ and Zn^2+^–Glu^–^ complexes. The M06-2X functional^41^ was employed for the DFT calculations. Geometry optimizations were performed at the M06-2X/6-31+G** level. Higher-level M06-2X/6-311++G(3df,2p) single-point energies were used to compute the binding free energies of various complexes at 298 K (Δ*G*_298_). The solvation effect of aqueous medium (ε = 78.4) was modeled with an implicit solvation model SMD.^42^

### 2.10 Application of Zinc−α-LA Complexes; Stability in Synthetic Physiological Fluids

Dissolution of zinc from the complexes was studied in four model synthetic fluids: gastric and intestinal with and without specific enzymes (pepsin and pancreatin for gastric and intestinal, respectively). The fluid was prepared as in ref (30). The sample was weighted on an analytical balance and dissolved in dissolution fluid. The solution was transferred to Amicon centrifugal device 3 kDa (Merck, Darmstadt, Germany). After 24 h of incubation time, the released zinc solution was separated by applying centrifugation (15 min, 14,000 rpm, 10 °C). The filtered solution was diluted (dilution factor 100) in 1% HNO_3_ (Merck Suprapure, Darmstadt, Germany). The quantitative analysis of zinc by the ICP–MS technique was performed as described in 2.4. Section.

### 2.11 Peptic Digestion Kinetics

For the study, the modified protocol from Pryshchepa *et al.*(43) was utilized. The digestion was performed in simulated gastric fluid prepared as follows: 2.0 g of NaCl was mixed with 80 mL of 1 M HCl and diluted to 1000 mL. The working pepsin (Sigma-Aldrich, Steinheim, Germany) solution with a concentration of 50 U/mL was prepared in simulated gastric fluid from stock solution (2000 U/mL in deionized water). The native α-LA and its complex with zinc were suspended in deionized water to a concentration of 10 mg/mL. The reaction mixture was prepared with an enzyme-to-substrate ratio of 0.5 U:100 μg by adding 80 μL of simulated gastric fluid, 10 μL of working pepsin solution, and 10 μL of protein or its complex solution to the Eppendorf tube. Final concentration of the protein in the solution was 1 mg/mL. Next, the mixture was incubated at 37 °C for 5, 15, 30, and 45 min. Termination of the reaction was performed by the addition of 0.7 M Na_2_CO_3_ at 35% of the reaction volume, that is, 35 μL was added to the reaction mixture. The control samples were prepared, where instead of protein/complex solution, 10 μL of reaction buffer was added to the reaction mixture. The control samples were incubated during 45 min. The SDS-PAGE analysis was performed in the reduced mode according to the procedure described in 2.1. Section with Perfect Color Protein Ladder (EURx Sp. z o. o, Gdansk, Poland) as a protein MW marker.

### 2.12 Biological Activity of Zinc−α-LA Composites

References (44)(45)–^46^ were utilized in developing this part of research (with some modifications). L929 and Caco-2 cell lines were purchased from ECACC (European Collection of Authenticated Cell Cultures operated by Public Health England) (Sigma). Both cell lines were cultured in DMEM supplemented with 10% (v/v) fetal bovine serum, 2 mM glutamine, 100 U/mL penicillin, and 100 μg/mL streptomycin (Sigma). The cells were passaged by trypsinization with 0.25% trypsin/EDTA every 3–4 days. For assays, cells were cultured on 96-well plates at a density of 2 × 10^5^ cells/mL and incubated for 24 h. When cells were attached to the bottom of the plate, the medium was replaced with a new one containing tested Ag complexes and incubated for 24 h. In control, the medium was replaced with a fresh one without tested substances. A silver nitrate control was performed to differentiate the cell response to various forms of silver compounds. After 24 h, 10% (v/v) of thiazolyl blue tetrazolium bromide (MTT) solution (5 mg/mL in PBS) was added to each well and incubated for 4 h at 37 °C. After that, medium from wells was removed, and the formazan crystals were dissolved in DMSO for 10 min by mixing. Absorbance was measured using a microplate reader (Multiskan, ThermoFisher) at 570 and 650 nm as background absorbance.

The LDH release assay was performed using a Lactate Dehydrogenase Activity Assay Kit (MAK066, Sigma). Cell cultures were prepared as for MTT assay, and cultured cells were incubated with the zinc complex and zinc nitrate to induce cytotoxicity and subsequently release enzyme lactate dehydrogenase (LDH). The medium with released LDH was transferred to a new plate and mixed with 50 μL of the reaction mixture. Absorbance was measured at λ = 450 nm using a multimode microplate reader (Varioskan TM LUX Thermo Fisher Scientific, Waltham, MA, USA). Results are presented as a percent of activity in comparison to the control.

The level of reactive oxygen species was measured with a Fluorometric Intracellular ROS kit (MAK144, Sigma-Aldrich). Cell cultures were prepared as for MTT assay, and cultured cells were incubated with the zinc complex and zinc nitrate to induce ROS for 24 h. After the incubation time, 100 μL of master reaction mix was added to each well and incubated for 30 min. The fluorescence intensity was measured at λ_ex_ = 540/λ_em_ = 570 nm using a multimode microplate reader (Varioskan TM LUX, Thermo Fisher Scientific, Waltham, MA, USA). Results are presented as a percent of activity in comparison to the control.

To check the amount of silver ions obtained from α-LA composites, L929 cells were incubated for 24 h with the Zn complex and Zn(NO_3_)_2_ at a concentration of 0.05 mM for 24 h. After that time, cells were washed two times with Dulbecco’s PBS, trypsinized with 0.25% trypsin, and again washed with Dulbecco’s PBS. The obtained cell pellet was mineralized with nitric acid.

## 3 Results and Discussion

### 3.1 Characterization of α-LA by SDS-PAGE

Figure S1 shows a gel electropherogram from α-LA analysis under reducing and nonreducing conditions. One single band occurs under these conditions. The band is around 14 kDa with respect to the standard markers. The initial use of concentrated protein solution (2 mg/mL) results in a wider band (lanes on the left side of the mode for reducing and nonreducing). The α-LA protein has a molar mass of about 14 kDa (from the amino acid sequence). The presence of one band in this region proves the purity and electrophoretic stability of the protein product. In particular, no band above 17 kDa for the marker was recorded. At this region of mass, it would come from the β-lactoglobulin, a protein which, next to α-LA, is a constituent of bovine whey proteins.^47,48^

### 3.2 Characterization of α-LA by the MALDI-TOF/TOF MS Technique

For further characterization of α-LA, mass spectrometry analyzes were performed using the MALDI technique. In order to obtain the molecular weight of the protein, the intact analysis was performed using the linear positive mode. Sinapic acid was used as a matrix while preparing the sample. Figure S2 shows the mass spectra. The most intense signal was recorded at an *m*/*z* of 14,166. This value indicates the α-LA adducts probably with hydrogen or sodium ions.^49^ A matrix and trifluoric acid are the source of these ions.^50,51^ The molecular weight value corresponds to the theoretical value for α-LA. Other peaks above the monomeric form can be distinguished. Peaks at *m*/*z*: 14,386, 14,496, and 14,599 correspond to other protein forms. Molar mass values of proteins will be differentiated by many factors (protein origin, post-translational modifications, and calcium content).^1,52,53^ The protein can be glycosylated at the asparagine moiety.^54,55^ Consequently, many isoforms can be characterized.^56,57^ The spectrum also shows a peak from the dimeric form occurring at *m*/*z* 28,326 and trimeric form (at *m*/*z* 42,495) and a low intensity peak from the doubly ionized form of the pseudo-molecular ion at an *m*/*z* of 7081. The choice of the matrix is crucial for observation and the stability of protein oligomers and isoforms under measurement conditions. The sinapic acid matrix can also distinguish oligomeric and isoform forms of β-lactoglobulin, while the HCCA matrix (another typical matrix used in proteomic studies) does not allow this. Jin and Manabe determined the molar mass of the monomer of α-LA for 14.18 KDa and discussed the effect of the residual content of foreign ions as impurities, especially sodium, and residual, nonwashed stain after unfolding the electropherogram, which have a measurable effect on the obtained results.^58^ Ham *et al.* determined the mass of the protein at around 14.2 kDa with an uncertainty of 4–105 Da for various samples prepared from Cow, Saanen, Toggenberg, and Alpine and found that the differences in the masses obtained were not significant.^59^ Svensson determined the molar mass at 14,088 kDa, and the difference between the determined and theoretical mass calculated from the amino acid sequence (14,078 KDa) attributed to post-translational modification (glycosylation and phosphorylation).^60^ In summary, our results are consistent with above-presented studies, and the differences in the determined masses result from (1) the origin of the protein, (2) post-translational modifications, and (3) residual impurities.

Protein identification was carried out by obtaining a unique peptide sequence after digesting the protein with trypsin. The corresponding peptide sequences are shown in Table S1. The resulting collection of peptides allows the identity of the protein to be established as α-LA.

### 3.3 Characterization of α-LA Saline Solution by Zeta Potential Determination

In order to investigate the stability of the α-LA solution in 0.09% NaCl (w/V), the zeta potential relationship was determined as a function of pH. The graph of this relationship is shown in Figure S3. The course of the dependence of zeta potential on pH is typical for a protein. We move from a solution in a strongly acidic environment where the measured zeta potential is strongly positive (above +20 mV) to an environment with higher pH values where the zeta potential approaches neutral values. A solution that exhibits a zeta potential absolute value greater than 25 mV is considered to be colloidally stable by the action of electrostatic repulsion between the colloid particles.^34^ In this case, it can be considered that the α-LA solution can be considered electrostatically stable only at the highest acidity of the solution. The protein shows a positive charge (it is positively electrostatically charged) due to protonation of amino acid functional groups. At pH 2, all amino acid residues are protonated initially. The amino acids with the most acidic properties in protein are the glutamic and aspartic acids. On the basis of the pKa values of these groups (3.22 and 2.77), at these pH values, exactly half of the content of these acids is deprotonated (neglecting the effect of the remaining amino acid groups on the p*K*_a_ value in α-LA). α-LA has 12 aspartic acid residues and 7 glutamic residues in its chain. This is a large proportion of acid functional groups in the protein structure, considering that α-LA has a total of 123 amino acid residues. At higher pH values, deprotonation begins to take place on amino acids with more and more basic properties (successively with polar and neutral side chain properties, acid amides, aliphatic moieties, and residues with basic side chain properties). The pH point at which the resultant of the positive and negative charges on the protein is zero is called the isoelectric point. This value for α-LA is 4.5 using the sigmoidal model.^61,62^ The charge of the protein, both dissociated and protonated groups, is equal to zero, and at this pH, we recorded the isoelectric point of the protein in a solution of 0.09% NaCl. The isoelectric point value is within the range of the literature values for α-LA. The value of the isoelectric point is a function of the composition of the solution.^48^ The solution under these conditions shows a number of properties: the protein is the least soluble, and the electrostatic repulsion between the individual groups of the protein is the lowest. This causes the protein under these conditions to have the greatest tendency to aggregate.^63^ The formed large particle clusters often sediment, and phase separation can occur. Moving toward the alkaline environment, the solution shows negative values of the zeta potential. They stabilize at around −25 mV at a pH of around 6.5. Under these conditions, the protein regains its electrostatic stability through ionization of individual functional groups of the protein. Relatively low values of the zeta potential deviation can be interpreted as an indicator of the lack of protein degradation, especially at extremely acidic and alkaline pH solutions, and consequently chemical stability to a harsh acid environment. However, the deviations arise with the acidity of medium. This trend of protein degradation by acid hydrolysis in very low pH will be visible particularly in dissolution studies of bound zinc cations in acidic artificial physiological fluid (Section 3.9).

### 3.4 Kinetic Study and Thermodynamic Data of Zinc Binding to Protein by the ICP–MS Technique

**Figure 1:**
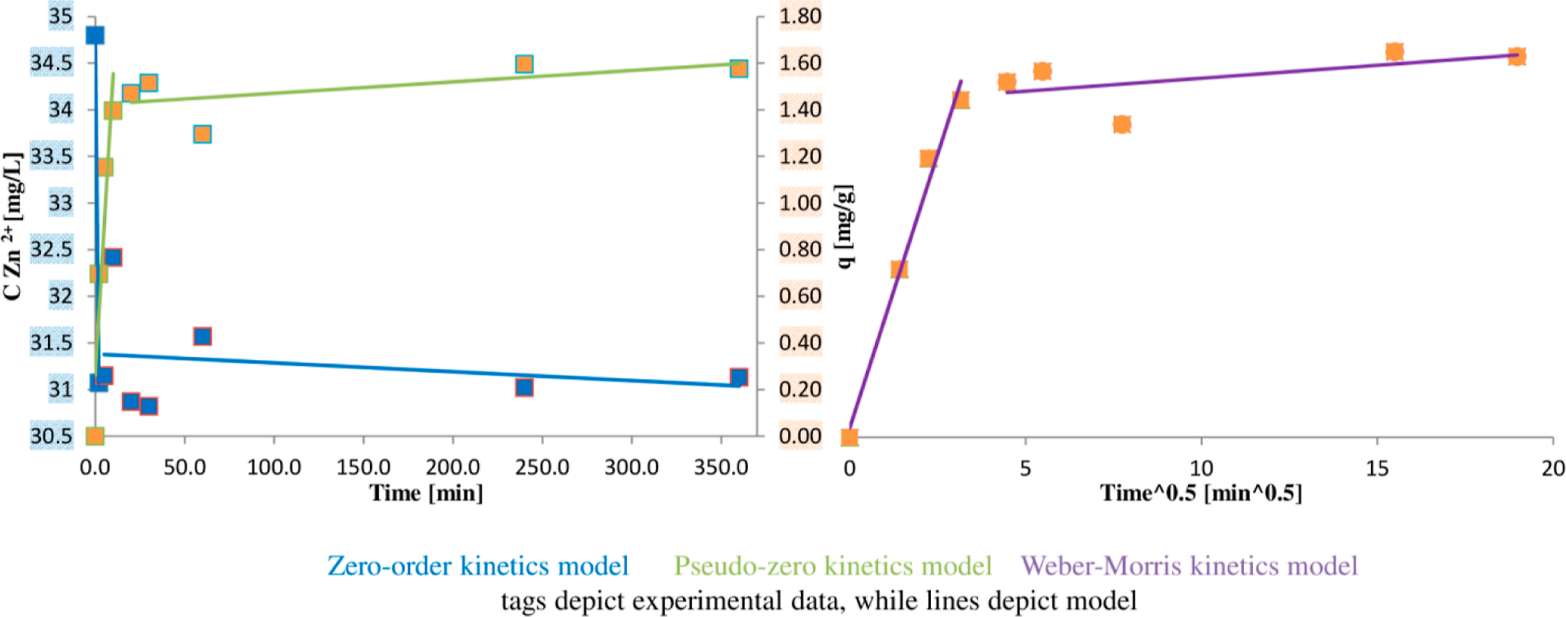
Zinc kinetics of binding to α-LA.

Figure 1 shows the dependence of unbound zinc concentration to α-LA as a function of the duration of kinetic experiment. Three different models of the fitting and interpretation of the results are presented: kinetic zero-order, pseudo-zero, and intraparticle diffusion models by Weber–Morris. Based on zero-order and pseudo-zero kinetic relationship, two stages of the ongoing process can be distinguished. The first stage is a rapid decrease in the concentration of zinc in the remaining solution. The second stage is leading to the stabilization of the concentration of zinc in the solution. The first stage is very quick. The reaction rate constant is 1.864 mg min per liter. Already, in the first measuring point (2 min of incubation), the concentration range is reached, which, based on the further course, can be called equilibrium. The second stage can be described as the stabilization and equilibrium stage, where the concentration of zinc in the solution fluctuates with respect to the equilibrium concentration. The reaction rate constant for this step (calculated with respect to the zero-order kinetic model) is equal to 7.3 × 10^–5^ mg min per liter. It can be concluded that the process of zinc binding by α-LA is very fast (after 2 min of incubation, the equilibrium is established), and the complexes were stable under the experimental conditions during the experiment. The intraparticle diffusion model is a fit of the experimental values in the system of coordinates of the adsorbed amount of zinc by the proteins to the root of the process duration. Experimental data show a gradual increase in the amount of zinc bound to the protein as a function of time. Stability in the recorded q values is visible at later measuring points. The Weber–Morris model is based on the linear dependence of q on the root of time. It is evident that the α-LA binding process for zinc is not linear for complete measuring time dimensions. Equilibrium values are shown; in particular, the *Q*_e_ value of 1.58 mg/g says that 1 g of protein binds 1.58 mg of zinc. The presented two models of matching the results allow concluding about the mechanism of the process. Based on the model of the zero-order kinetics, it can be stated that the process is fast, the rate-limiting stage is the diffusion of zinc cations to the surface of the protein structure, and the functional groups are available on the surface. It should be emphasized that the process is located on the surface. There are no additional steps of zinc binding through α-LA (a symptom would be a further, stepwise decrease in the recorded concentration in the remaining solution or an equilibrium increase in the q value). In other words, no further diffusion of zinc cations into the interior of the protein structure is observed. Detection of a further stepwise increase in the amount of zinc by the protein would be visible using the Weber–Morris model.^64^

The amount of bound zinc by protein under equilibrium conditions ranged from 30.05 mg/g for casein mixtures, from 5.16 to 6.85 mg/g for individual forms of casein, and 8.16 mg/g for bound zinc by beta-lactoglobulin based on studies performed in our research unit.^30,32,33^ It can be seen that the α-LA protein does not show much value of binding zinc ions from the solution. This may be due to the availability of zinc binding sites for the holo protein. In addition, it is known that the sites are located on the surface, and the experiment was conducted under the pH conditions at the isoelectric point, where accessibility to the surface is the lowest. Buszewski *et al.* also noted the effect of the molar mass of a protein on the value of the bound portion of the metal by the protein. The smaller the protein, the lower the possible availability of appropriate functional groups and, consequently, the number of active centers capable of interacting.^30^ Hence, α-LA protein is the smallest protein, in terms of molecular weight, tested in our research group. α-LA has a numerically low content of functional groups that are able to interact with zinc ions compared to heavier proteins, which have far more active functional groups to absorb cations. The influence of the process conditions through the prism of zinc cations and possibly the influence of conformation of the zinc aqua complex and charge localized on the surface (which is positive in the pH of the process) occur. The process at pH 4.5 determines the value of the electrostatic attraction force in relation to the protein structure. The net charge of α-LA is zero, but the active functional groups (aspartic and glutamic acids in particular) are fully deprotonated and negatively charged. In addition, the pH value of the isoelectric point determines the conformation of the protein, where the presence of polar groups available for interaction is the highest due to the hydrophobic effect occurring at around pH of isoelectric point of protein. Thus, the process binding of zinc for α-LA is surface-localized only. Table 1 shows the thermochemical data from the kinetic experimental data. The value of the Gibbs enthalpy is negative. Thus, reaction has a spontaneous tendency to occur. There is a visible correlation between the quoted other protein studies tested in our team and the values of Gibbs enthalpy for the study of zinc binding. Gibbs enthalpy is lower for heavier proteins compared to α-LA. Great importance here is the entropy effect, which assumes more favorable changes from the thermochemical point of view for these systems, where, as a result, a more disordered system is formed. This can manifest itself in conformation changes, ion-exchange reactions,and so forth*.*

**Table 1:** Thermodynamic Parameters Obtained from the Kinetic Study of Zinc Binding to α-LA

parameters	value [units]
*C*_e_	3.7 [mg/L]
*Q*_e_	1.58 [mg/g]
*K*_D_	47.3
Δ*G*	–9.45 [kJ/mol]

### 3.5 Characterization of α-LA Complexes with Zinc by Spectroscopic Techniques

**Figure 2:**
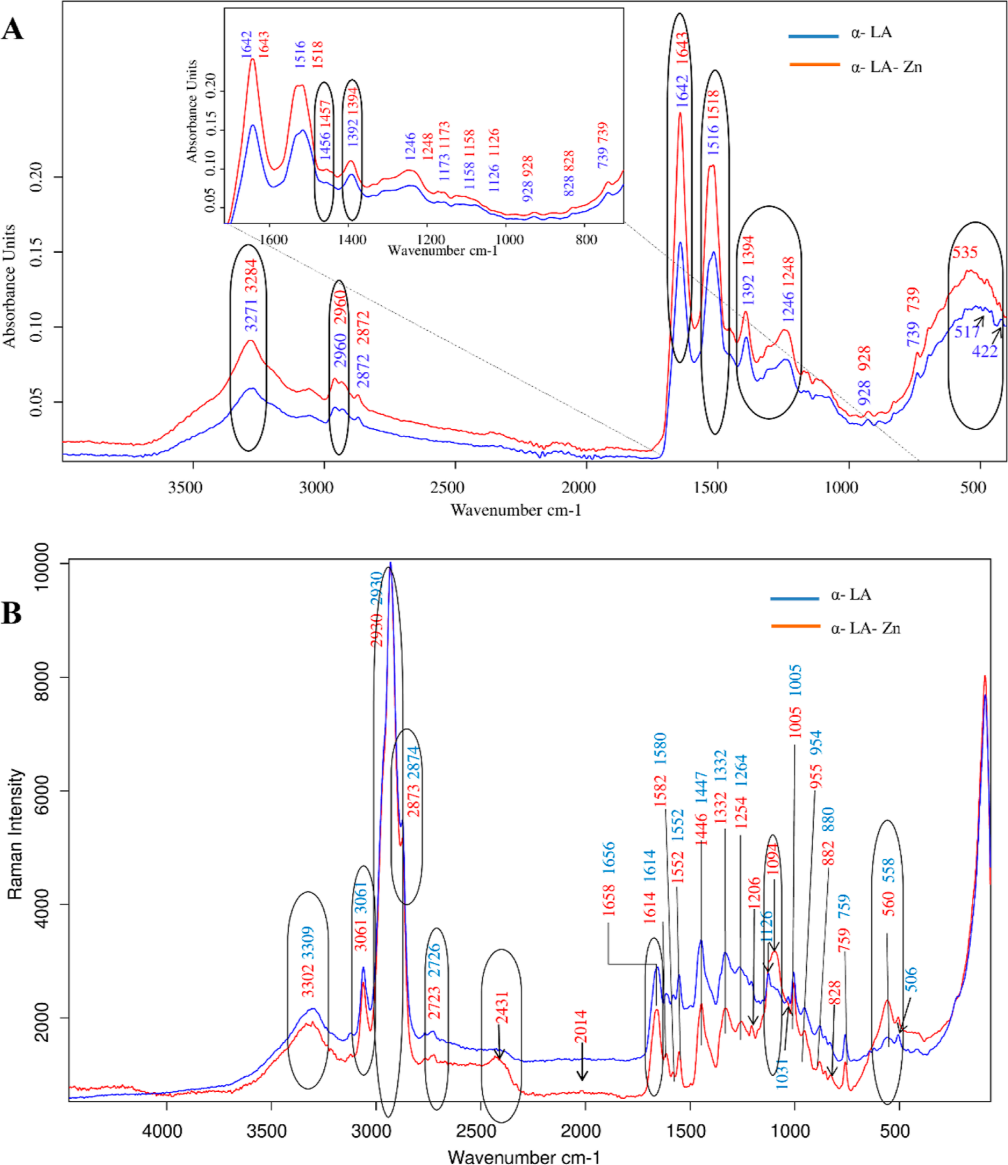
(A) ATR-IR spectrum of α-LA (blue line) and α-LA complexes with zinc (red line); (B) Raman spectrum of α-LA (blue line) and α-LA complexes with zinc (red line).

Figure 2A shows the infrared spectra of the α-LA protein (as a control) and α-LA complex with zinc ions. Noteworthy is the fact that selective increases in absorbance for specific vibrations took place for α-LA complexes with zinc compared to the control. In the beginning of interpretation from the highest wavelength region, the first bands at 3271 cm^–1^ (control) and 3284 cm^–1^ (α-LA–Zn) correspond to the vibration of the amine group N–H of amide I.^65^ Changes in the frequency of stretching vibrations (N–H) in this respect between control and complex compounds are the result of changes in the values of the share of hydrogen bonds. The consecutive bands located at lower frequencies are responsible for the stretching vibrations of the aliphatic C–H and also of the amine groups. Another range in which the vibration bands are observed is the vibration range of amide I. In this range, vibrations from the carbonyl group C=O have their very strong band (1642 and 1643 cm^–1^ for control and complexes, respectively).^66^ Another band can be attributed to the vibration of amide II. The N–H and C–N groups have their vibrations here. Two consecutive bands present at about 1456 and 1392 cm^–1^ are responsible for the C–H bending vibration of the amino acid groups. The next bands are the vibrations of the amide III group coming from the N–H bending and C–N stretching vibrations. Subsequent bands come from the vibrations of aromatic amino acid groups,^67^ unless there were any drastic changes in the IR spectrum of the α-LA and after the formation of complexes with zinc, which is consistent with the literature data. The greatest changes in the distribution of the bands were noted for the region with the lowest vibration frequencies. This region, from aromatic vibrations, and the environment around these groups probably change (*e.g.*, conformational).^68^ The region of the participation of individual conformations (ρ-helix, β-sheet, β-turns, and random coil) can be attributed to the vibration of amide III.^69^ Especially, in the enlargement of the fingerprint region, there is a visible change in the relative intensity of the vibration between the native structure and the binding to zinc. Thus, the protein conformation change after binding with zinc is very possible. After the binding of the protein with the zinc, a band at 535 cm^–1^ was visible, while before the binding, bands at 517 and 422 cm^–1^ were visible.

Figure 2B shows the Raman spectrum of the α-LA protein before binding and its complex with zinc. Similar to the IR technique, Raman is a complementary technique, which, unlike IR, especially shows vibrations from groups with a low difference in electronegativity in the group, while IR shows vibrations of ionic groups in particular. Also starting the description from the bands at the highest frequencies toward the lower ones, the first band at around 3300 comes from the stretching vibration of the N–H amino group. A clue that it is a vibration of the amino group may be the intensity of the vibration, which we judge as an average. Vibrations of other groups with a similar range (O–H or =C–H) have a weak intensity in the Raman spectrum. The change in the frequency of this protein oscillation after binding may also be the result of changes in the orientation of the hydrogen bond. The next bands at 3061, 2930 cm^–1^, and around 2873 cm^–1^ are the vibration bands of =C–H and C–H, respectively. The bands at around 2723 and 2431 cm^–1^ are responsible for S–H vibrations, while after the binding of zinc to the protein, the second band is not visible.^70^ The band at around 1658 cm^–1^ is responsible for amide I vibrations. The carbonyl group of glutamic and aspartame amino acids gives the band from the C=O stretching vibration.^71^ Then, the spectra show a rich set of bands derived from vibrations of individual amino acid groups (fingerprint region).^71,72^ The two bands were significantly enhanced in intensity compared to the control. The bands at 1094 and 560 can be attributed to C–S aliphatic and aromatic vibrations, respectively.^70,72^

### 3.6 Characterization of α-LA Complexes with Zinc by Microscopic Techniques

**Figure 3:**
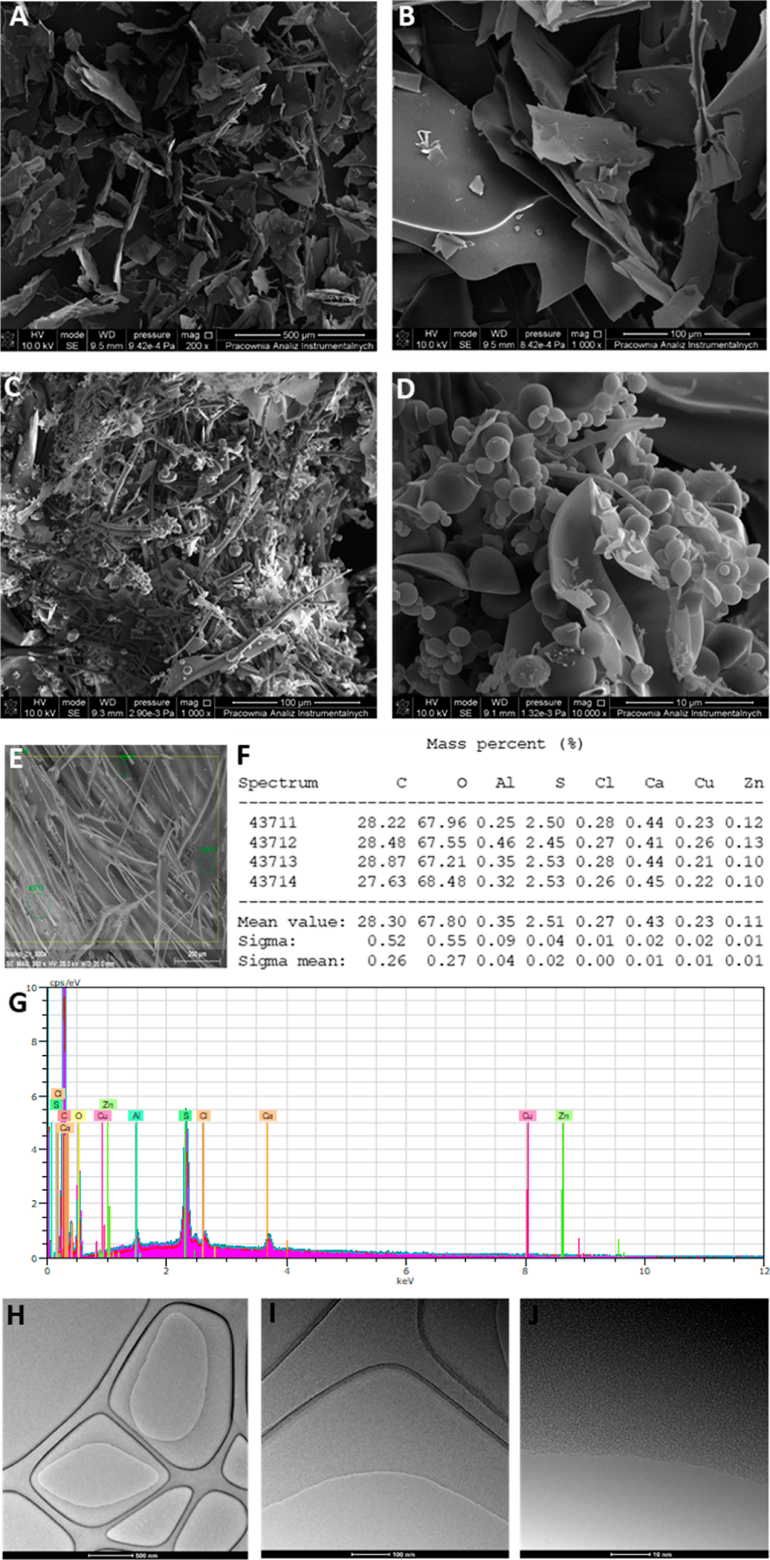
(A,B) SEM pictures of the α-LA protein with different magnifications of the sample place; (C–E) SEM pictures of the Zn−α-LA complex with different sample place magnifications; (F) SEM–EDX imaging, photograph of the sample; (G) spectrum from the selected image; (G) list of characterized elements with the content of individual elements in the material; and (H–J) TEM pictures of the Zn−α-LA complex with different bars.

Figure 3A,B shows the results of surface imaging and examination of the surface morphology of α-LA protein by the SEM method. The surface is flat and continuous without visible bulges, flooding,and so forth*.* The petals rarely show small clusters of particles that can be assessed as impurities. Figure 3C,D shows the characteristics of the Zn complex with α-LA. After the zinc binding process by α-LA, visible changes took place on the surface. When assessing the photograph with the smallest magnification, we can see clusters of particles that are significantly whiter and brighter (Figure 6C). This effect can be attributed to the bound zinc ions on the surface of the metal–protein composite. Additionally, it can be assessed that these places are located rather evenly, without segregation. In addition, the metal deposit is rather in the surface layers, with a visible scaly structure from the protein in the deeper layers of the material. Moving on to photographs with larger enlargements, the surface structure is visible. There are numerous particles in the shape of spheres, tightly adhering to the surface of the protein. The whiter shade of these beads as mentioned earlier is due to the zinc-rich material. The deposit globules adhere tightly to each other, forming large clusters which, when enlarged further, appear as a compact surface. Image 6E shows a low-resolution image taken during EDX imaging. It shows a dense, compact structure. From the marked area, spectra of the selected elements were collected. The image 6F shows the spectra of individual elements. In addition to zinc, which is evenly distributed (low Sigma value) (Figure 3G), there are other elements coming from the protein, the mesh in which the sample was placed (Cu) and typical contamination of the sample from, for example, water (Cl).

Figure 3H,I,J shows the TEM image of the material after the zinc binding process by α-LA. Each image shows an amorphous form of the material in greater zoom. There are no visible clusters or clumps of particles that could indicate the formation of zinc oxide nanoparticles on the surface of the protein or the precipitation of zinc to a metallic form.^73^ On this basis, it can be concluded that the process of zinc binding to α-LA proceeds in accordance with the electrostatic attraction of zinc ions to the oppositely charged functional groups of the protein, without the charge transfer process (oxidation or reduction).^74^

### 3.7 Characterization of α-LA Complexes with Zinc by MD Simulations

**Figure 4:**
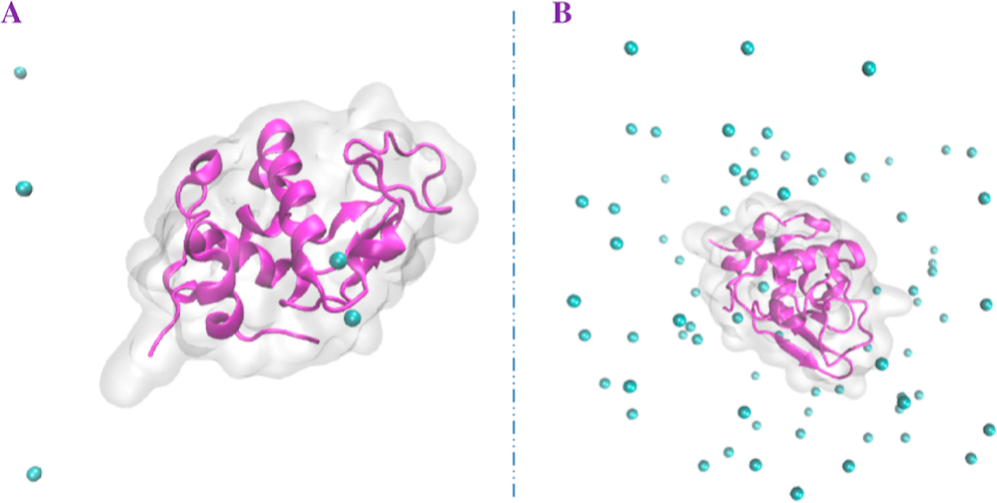
Modeled structures: α-LA with its apo form (A) and modeled complex with zinc cations (with a concentration of 30 mg Zn/L in the reagent mixture) (B).

Figure 4 depicts the modeled structure of protein and the complex with zinc cations with concentration used in all studies (30 mg Zn/L). These structures were obtained after the energy minimization procedure.

Table 2 shows the percentage of participation of amino acid residues in interaction with zinc. Analyzed interactions between Zn cations and GLU, ASP, CYS, HIS, TYR, TRP, PHE, MET, ARG, and LYS with a distance threshold of 0.35 nm were examined. Glu and Asp amino acids are dominating with interaction with zinc cations, while smaller contribution has lysine with interaction.

**Table 2:** Participation of Amino Acids of α-LA in Interaction with Zinc Cations at Studied Concentration of Metal

AA	1A4V–Zn^2+^ (39) n(bind. sites)	1A4V–Zn^2+^ (39) %(bind. sites)
GLU	**830**	**43.252**
ASP	**994**	**51.798**
CYS	4	0.208
HIS	0	0.000
TYR	4	0.208
TRP	0	0.000
PHE	3	0.156
MET	9	0.469
ARG	0	0.000
LYS	**21**	**1.094**
	**1865**	

**Figure 5:**
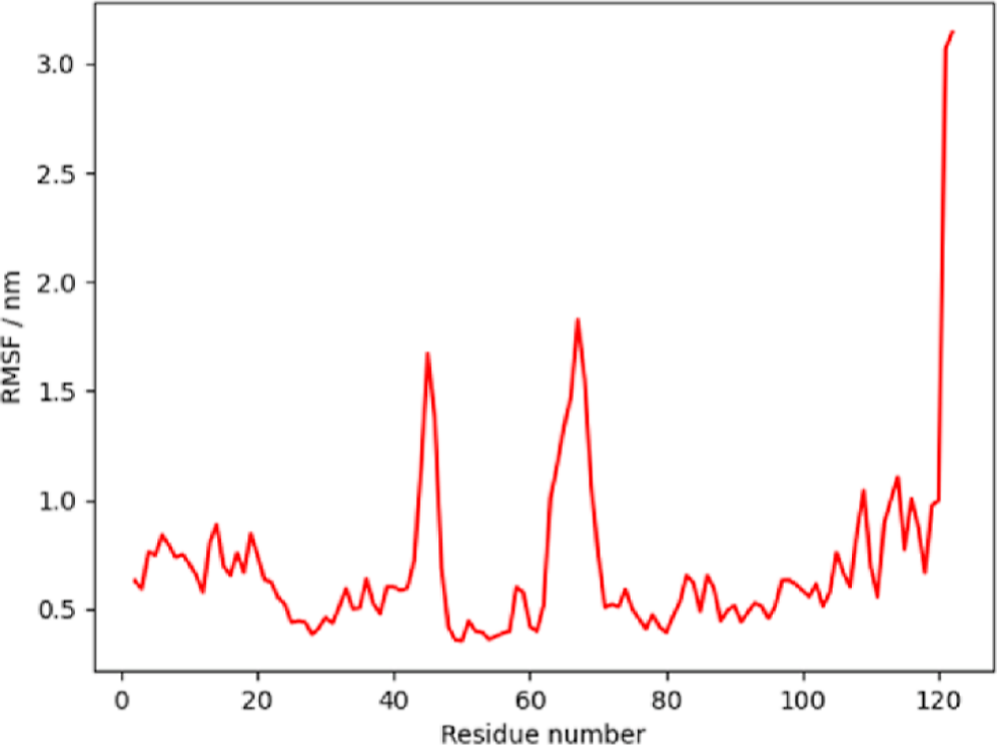
Flexibility analysis of α-LA–Zn complexes.

The flexibility of the four key regions decreases with the increase in zinc concentration. At around residues 15–20, 30–50, 60–80, 100–120, 15–20: 3 turn-helix (310), 30–50: hydrogen-bonded turn, extended strand (beta sheet), 60–80: same as 30–50 + *a* 3-turn helix, 100–120: four-turn helix (alpha), three turn-helix and random coil were detected (Figure 5).

### 3.8 Binding Interactions of Aspartate and Glutamate Residues toward Zn^2+^ (aq)

**Figure 6:**
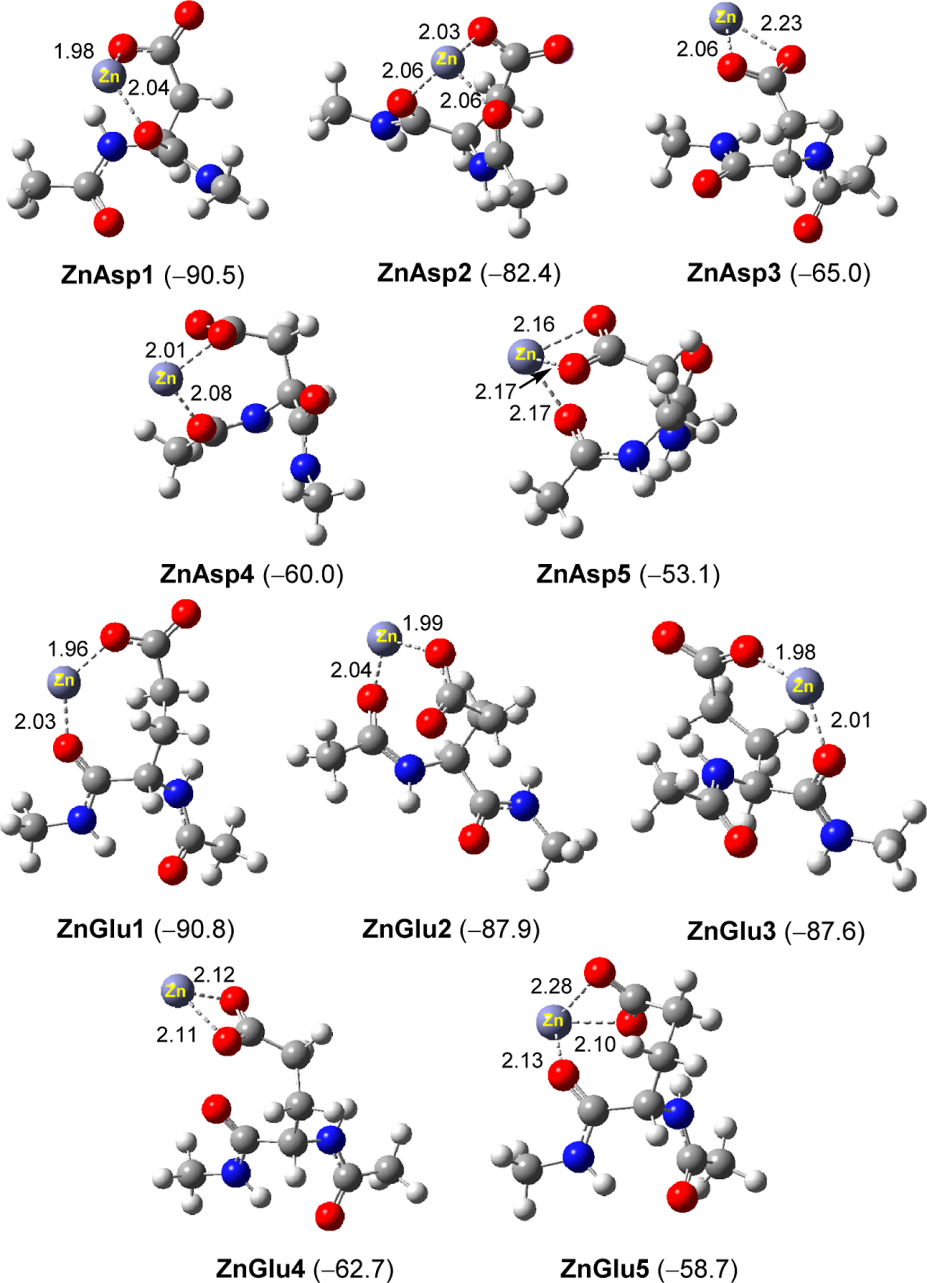
Optimized geometries of various conformations of Zn^2+^–Asp^–^ (**ZnAsp1**–**ZnAsp5**) and Zn^2+^–Glu^–^ (**ZnGlu1**–**ZnGlu5**) complexes. Interaction distances are in Å, and binding free energies (Δ*G*_298_) are in kJ/mol.

The MD simulations have demonstrated the dominant occurrence of aspartate and glutamate residues, which indicated that the negatively charged Asp^–^ and Glu^–^ residues are the strongest Zn^2+^ binders. DFT calculations were performed to further shed light on the possible binding modes and interaction energies of Zn^2+^ with aspartate and glutamate residues. To this end, various possible conformations of 1:1 Zn^2+^–Asp^–^ and Zn^2+^–Glu^–^ complexes were investigated. The Asp^–^ and Glu^–^ residues were modeled with two units of amide linkage and capped with methyl groups in order to investigate the interaction of Zn^2+^ with the protein polypeptide backbone. The solvation effect in an aqueous environment was modeled using the implicit SMD solvation model. The optimized geometries and binding free energies (Zn^2+^ + AA^–^ → Zn^2+^–AA^–^, Δ*G*_298_) of the five lowest energy conformations of Zn^2+^–Asp^–^ and Zn^2+^–Glu^–^ complexes (**ZnAsp1**–**ZnAsp5** and **ZnGlu1**–**ZnGlu5**, respectively) are summarized in Figure 6. As expected, mondentate/bidentate interaction between the Zn^2+^ cation and the negatively charged carboxyl group (COO^–^) represents the key interaction, which readily attributed to the strong electrostatic attraction between Zn^2+^ and COO^–^. Simultaneous coordination of Zn^2+^ with the carboxyl group of the backbone, *via* carbonyl oxygen, is observed in most complexes. The Zn^+^···O (carbonyl) interaction distances, 2.01–2.17 Å, are comparable to those in Zn^2+^–COO^–^ interactions, 1.96–2.28 Å. Several tri-coordinated Zn^2+^ complexes, namely, **ZnAsp2**, **ZnAsp5**, and **ZnGlu5**, were observed. However, these conformations are less stable due to the unfavorable entropy effect. For both Zn^2+^–Asp^–^ and Zn^2+^–Glu^–^ complexes, the lowest energy conformation, namely, **ZnAsp1** and **ZnGlu1**, respectively, corresponds to a coordination geometry with the zinc ion two-coordinated with carboxyl and carbonyl (side chain) groups. The calculated binding free energies (Δ*G*_298_) of various conformations of both Zn^2+^–AA^–^ complexes fall in the range −53.1––90.8 kJ/mol (Figure 6). This indicates that the formation of the Zn^2+^–AA^–^ complex is energetically favorable. The calculated strong binding affinity supports the experimental observation of the interaction of Zn^2+^ ions with aspartate and glutamate residues of α-LA protein.

### 3.9 Application of Zinc−α-LA Complexes; Stability in Synthetic Physiological Fluids and Biological Activity

This section of research focuses on contents of zinc released from the complex under four different conditions simulating the stomach and intestine conditions. About 10% of zinc, which was initially bound to the protein, was released in an acidic environment. No effect of the enzyme was noted. The pepsin was unable to cause the effect of dissolving zinc from the complex and degrading the protein into short peptides that could pass into the solution tested under the conditions of this experiment. In the alkaline environment without the participation of the enzyme, the release value of less than 10% was also noted, which confirms the stability of the complex to the alkaline environment. However, the action of this alkaline medium together with the pancreatin enzyme causes the release of a significant amount of zinc (41.4%), and consequently, it can be assessed that the complex is not stable under these conditions. Rodzik *et al.* studied the release of zinc from complexes with β-lactoglobulin.^30^ The stability of the complex in both environments (gastric and intestinal fluid) acting on the complex without the enzyme is noted. However, along with the enzymes, zinc was released from the complex. In this case, the complex is insensitive to the enzyme acting in the acidic medium, unlike the studies in ref (30). As in the studies by Rodzik *et al.*, the influence of the pancreatin enzyme in the intestinal fluid was noted here. This may be due to the action of the enzyme trypsin, which degrades protein into peptides, which is part of the release solution.^30^

**Figure 7:**
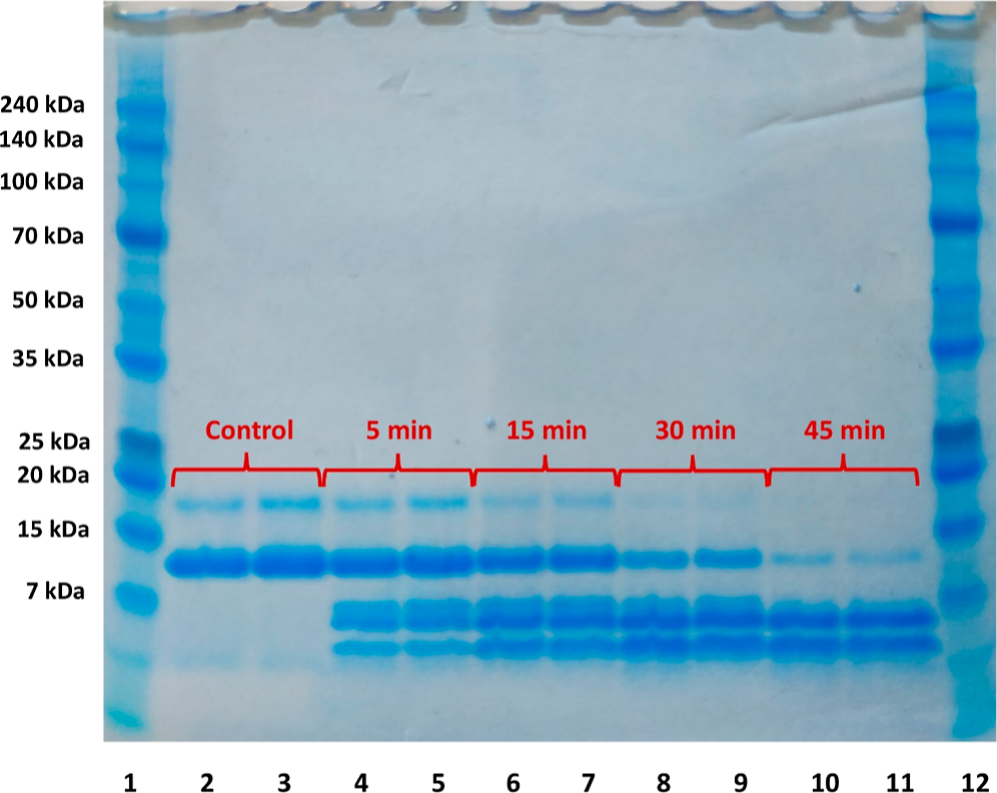
SDS-PAGE presenting peptic digestion kinetics, where bands of native α-LA are presented on 2, 4, 6, 8, and 10 and the bands of the complex of α-LA with zinc are presented on 3, 5, 7, 9, and 11.

Furthermore, the peptic digestion kinetics was performed to visually demonstrate the digestive stability of the synthesized complex. Figure 7 presents the resulting SDS-PAGE for performed digestions.

The results of the peptic digestion revealed that complete α-LA hydrolysis was not achieved even after 45 min. Instead, in the previous work of our group, the kinetics of bovine lactoferrin (bLTF) digestion was performed. After 30 min, there were no intact bLTF in the solution,^43^ while almost 50% of α-LA at this time point still was unchanged. It is noteworthy to mention that for bLTF digestion, a lower enzyme-to-protein ratio was utilized (0.1 U/100 μg) which indicates much higher susceptibility to peptic hydrolysis. The proteins’ digestion susceptibility with enzymes is dependent on the structure of the protein. More tightly folded sequences reveal lower susceptibility to enzymatic degradation.^75^ bLTF has a hinge region connecting N- and C-lobes which seems to be the most preferable place for the enzymatic action. Moreover, it was reported that bLTFs’ N-lobe treated with pepsin can easily release antibacterial peptide lactoferricin. Additionally, the digestion appears more intense upon the loss of the iron from the structure which occurs under acidic conditions.^76^ Instead, α-LA under acidic conditions forms a highly stable molten globule which may be a reason for its increased stability against peptic digestion.^77^ The digestion kinetics of the complex of α-LA with zinc did not differ from the kinetics of native protein. Interestingly, the peptic digestion of α-LA cause the formation of three peptides with masses lower than 7 kDa, and two of them remain unchanged even after 45 min of the process. It may be concluded that zinc from the complex should remain bonded to these peptides. Instead, for the complex of bLTF with Ag, a slightly lower degradation rate for the protein was observed, but much faster digestion of peptides occurred. The respective differences may be due to the changes in the tertiary structure caused by Ag incorporation.^43^ It is noteworthy to mention that in Permyakov *et al.* work,^27^ the tryptic digestion of α-LA was performed in the presence of Zn^2+^ ions. They have utilized both the trypsin and chymotrypsin for the digestion. It was shown that in the case of trypsin, the presence of Zn^2+^ increases the digestion rate in all utilized Zn/protein ratios. Instead, for chymotrypsin, the acceleration began at somewhat higher metal concentration. The differences may be connected to the differences in their specificity: trypsin cleaves at peptide bonds containing basic residues, while chymotrypsin cleaves at peptide bounds adjacent to aromatic residues. Pepsin is a protein that also preferentially hydrolyzes peptide bonds between the aromatic amino acids which may explain the observed results as the batch sorption analysis did not show the high Zn^2+^ sorption by α-LA.^78^

Cytotoxicity of Zn−α-LA composites and Zn ions was determined by MTT and LDH methods on human epithelial colorectal adenocarcinoma Caco-2 cell lines and L929 murine fibroblast cell lines. L929 cells are used in ISO 10993-5 and ISO 10993-12 norms for biological and clinical evaluation of medical devices. Caco-2 cells, due to many morphological and biochemical similarities to enterocytes—intestinal absorptive cells, are used as *in vitro* models to study absorption of orally administered drugs. Cell viability was tested using two spectrophotometric assays: MTT (3-(4,5-dimethylthiazol-2-yl)-2,5-diphenyl tetrazolium bromide) and LDH (lactate dehydrogenase) release. MTT test is based on the ability of mitochondrial enzyme dehydrogenase to transfer yellow tetrazolium dye into formazan crystals. The level of obtained formazan crystals is directly proportional to cell viability. Therefore, the level of formazan crystals in the control, untreated sample is set to 100% viability. In the LDH assay protocol, the level of lactate dehydrogenase that is released into the culture medium following the loss of membrane integrity is measured. LDH activity is recognized as an indicator of cell membrane integrity.

**Figure 8:**
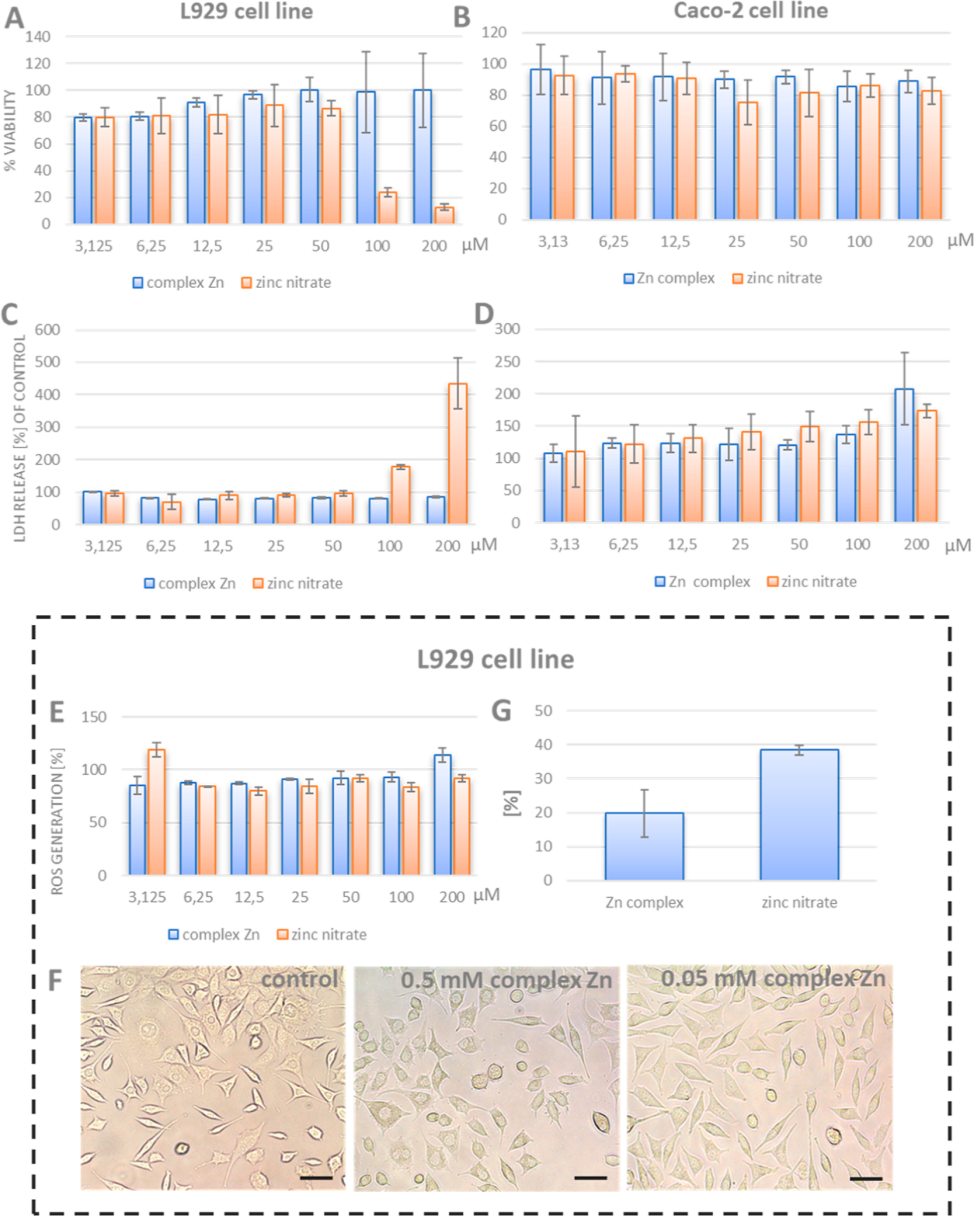
(A,B) L929 and Caco-2 cell viability after treatment with the Zn complex and Zn ions measured by the MTT method; (C,D) LDH leakage level of L929 and Caco-2 cells treated with Zn ions and the Zn complex; (E) concentration-dependent ROS generation by Zn ions and the Zn complex in L929 cells; (F) morphology of L929 cells treated with the Zn complex; and (G) % of silver taken up by L929 cells from the Zn complex and zinc nitrate. For (A–E), * indicates statistically significant differences (*p* ≤ 0.001) between the sample and control according to one-way ANOVA and the Tukey post hoc test. For (G), * indicates statistically significant differences (*p* ≤ 0.001) between the uptake of zinc from the Zn complex and zinc nitrate according to one-way ANOVA and the Tukey post hoc test.

As shown in Figure 8A,B, the MTT results demonstrated that in L929 cells, Zn in the form of complex did not decrease the viability of cells in the whole range of tested concentrations, that is, up to 200 μM. However, zinc ions were more toxic and lowered the viability of cells to 20% at a concentration of 100 μM. Caco-2 cells were less sensitive to both forms of Zn than L929 cells. One of the reasons for the difference may be that L929 cells belong to the normal cell line, while Caco-2 cells are from malignant tissue, colorectal adenocarcinoma.

To monitor the membrane damage of more susceptible L929 cells, LDH test was performed (Figure 8C,D). The results of this assay show that the lactate dehydrogenase release is mostly very similar for zinc complexes in the whole range tested and comparable with the control. For zinc ions, a significant increase in the dehydrogenase released was observed at the concentration 200 μM, which indicates damage to the integrity of the membrane. For cells, Caco-2 levels of released lactate dehydrogenase were only slightly elevated in the range 6.25–200 μM.

One of the aspects of cytotoxicity is oxidative stress. In order to detect reactive oxygen species after treatment with zinc ions and complexes, a fluorometric intracellular ROS kit that detects, in particular, superoxide and hydroxyl radicals was applied (Figure 8E). Studies showed that the level of ROS for cells treated with zinc ions and zinc complexes was comparable with control cells. Also, a comparison of the morphology of cells treated with various concentrations of zinc complexes did not reveal any significant changes compared to control cells.

To check the amount of zinc ions taken from the protein complexes, L929 cells were incubated for 24 h with zinc complexes and for comparison with zinc nitrate (Figure 8G). Results showed that the level of adsorbed zinc was two times higher for zinc nitrate than for zinc complexes. However, many studies indicate that at higher zinc concentrations, zinc uptake is by passive diffusion (for review, 68). Lower values of zinc taken by cells from zinc complexes indicate another, more safe mechanism of zinc absorption. However, further studies especially on Caco-2 cells are necessary for understanding the transport mechanism of zinc complexes.

## Conclusions

This article presents the physicochemical characteristics of α-LA and the synthesis of the complex of the protein with zinc ions. The binding process was investigated with several analytical techniques. The work shows that the binding process is fast and zinc ions are bound to the surface of protein particles in solution, while aspartic and glutamic acids are particularly active functional groups in metal ion binding. The solubility, bioavailability, and cytotoxicity of these complexes were also tested.

## Supporting Information Available

SDS-PAGE analysis of the α-LA standard; mass spectra of α-LA recorded in the linear positive mode by MALDI-TOF MS; identified peptide sequence characteristic for α-LA, protein recorded with MALDI-TOF MS in the collision mode after digestion reaction with trypsin; and pH dependence of zeta potential for protein suspension in 0.09% (w/V) NaCl (PDF)
