# Supplementary material for: Functionalization of Alpha-Lactalbumin by Zinc Ions
Source: ACS Omega. 2022 Oct 21;7(43):38459–74. doi: 10.1021/acsomega.2c03674 (PMC9631873; doi:10.1021/acsomega.2c03674)
Supplement: Supplementary file 1 — ao2c03674_si_001.pdf [file ao2c03674_si_001.pdf]

Supporting Information

Functionalization of alpha-lactalbumin by zinc ions

Adrian Gołębiowski[1,2], Paweł Pomastowski[1*], Katarzyna Rafińska[2], Petar Zuvela[3], Ming Wah Wong[3], Oleksandra Pryshchepa[1,2], Piotr Madajski[4], Bogusław Buszewski[1,2]

[1] Centre for Modern Interdisciplinary Technologies, Nicolaus Copernicus University in Torun, 4 Wileńska St., 87-100 Torun, Poland
[2] Department of Environmental Chemistry and Bioanalytics, Faculty of Chemistry, Nicolaus Copernicus University in Torun, 7 Gagarina St., 87-100 Torun, Poland
[3] Department of Chemistry, National University of Singapore, 3 Science Drive 3, 117543, Singapore
[4] Department of Chemistry of Materials Adsorption and Catalysis, Faculty of Chemistry, Nicolaus Copernicus University in Torun, Gagarina 7, 87-100 Torun, Poland

[*] Correspondence: p.pomastowki@umk.pl

Contents

Figure S1. SDS-PAGE of α-LA. The reduced and non-reduced conditions were applied. Three 10X serial dilution was analyzed. 2

Figure S2. Mass spectrum for α-LA obtained in Intact mode of MALDI-TOF-MS spectrometer. 3

Table S1. MS and MS/MS identification of α-LA peptides. 4

Figure S3. Zeta-potential dependence of α-LA on the pH in a saline solution. 5

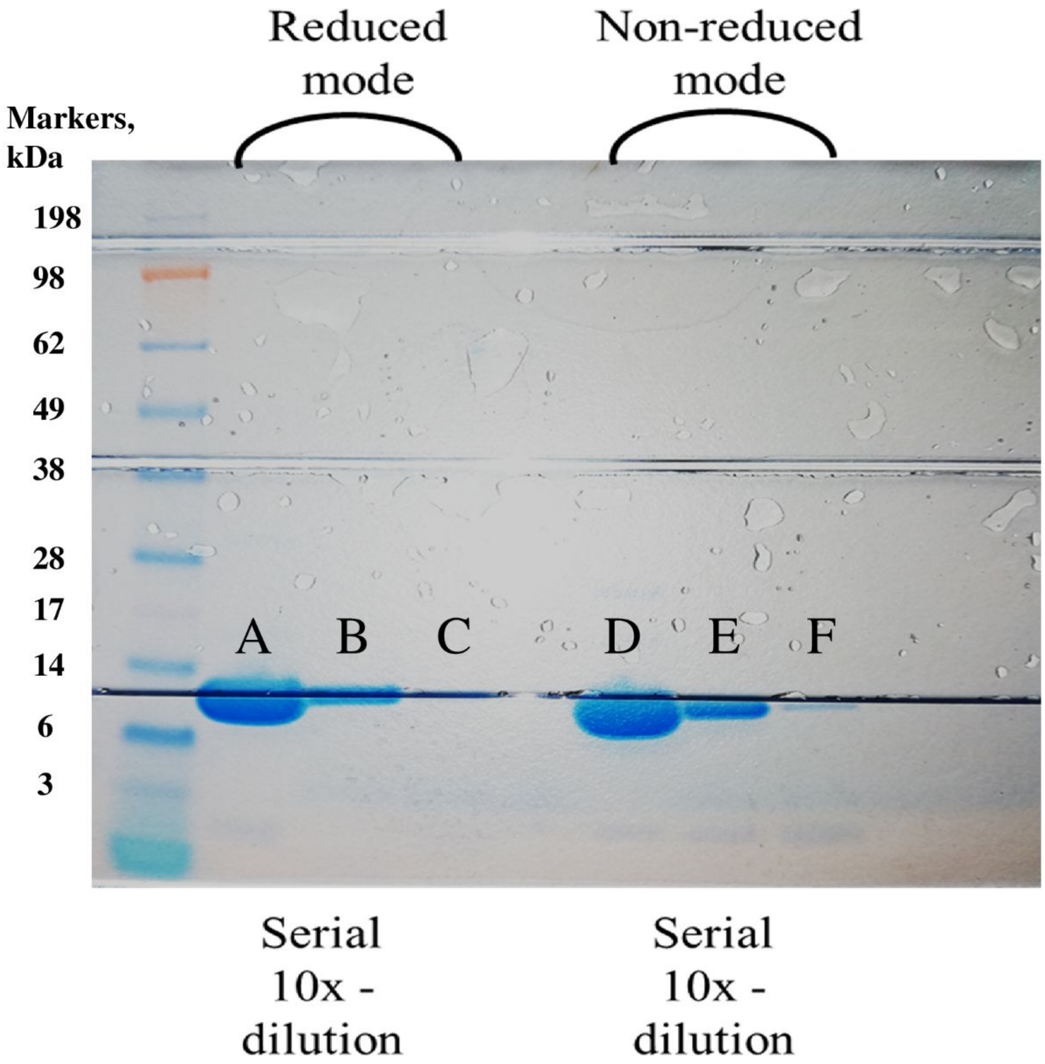

Figure S1. SDS-PAGE of α-LA. The reduced and non-reduced conditions were applied. Three 10X serial dilution was analyzed.

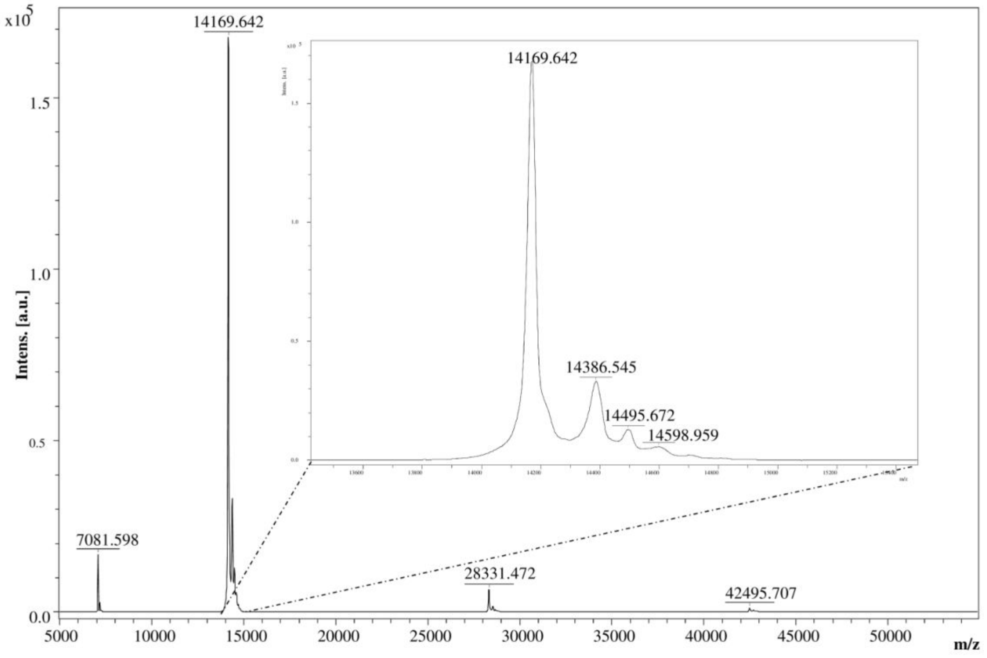

Figure S2. Mass spectrum for α-LA obtained in Intact mode of MALDI-TOF-MS spectrometer.

Table S1. MS and MS/MS identification of α-LA peptides.

Mass [Da]		Intensity	Sequence Range	Sequence from MS/MS
Measured	Theoretical			
710.330	710.329	33013.490	25 - 29	CEVFR
1091.520	1091.519	4357.889	134 - 141	LDQWLCEK
1200.666	1200.652	203435.100	118 - 127	VGINYWLAHK
1699.774	1699.755	4108.163	99 - 112	FLDDDLTDDIMCVK
2003.818	2003.818	1341.993	82 - 98	DDQNPHSSNICNISCDK

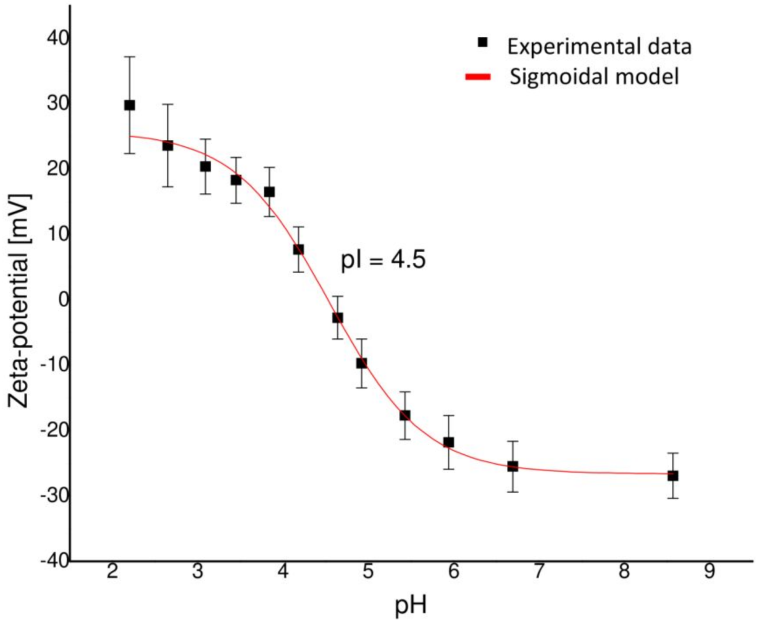

Figure S3. Zeta-potential dependence of α-LA on the pH in a saline solution.
